# Diabetes, Obesity, and Hypertension May Enhance Associations between Air Pollution and Markers of Systemic Inflammation

**DOI:** 10.1289/ehp.8469

**Published:** 2006-02-27

**Authors:** Sara D. Dubowsky, Helen Suh, Joel Schwartz, Brent A. Coull, Diane R. Gold

**Affiliations:** 1 Department of Environmental Health and; 2 Department of Epidemiology, Harvard School of Public Health, Boston, Massachusetts, USA; 3 Channing Laboratory, Department of Medicine, Brigham and Women’s Hospital, Boston, Massachusetts, USA; 4 Department of Biostatistics, Harvard School of Public Health, Boston, Massachusetts, USA

**Keywords:** air pollution, C-reactive protein, inflammation, metabolic syndrome, particulate matter, susceptibility

## Abstract

Airborne particulate matter (PM) may lead to increased cardiac risk through an inflammatory pathway. Therefore, we investigated associations between ambient PM and markers of systemic inflammation among repeated measures from 44 senior citizens (≥ 60 years of age) and examined susceptibility by conditions linked to chronic inflammation. Mixed models were used to identify associations between concentrations of fine PM [aerodynamic diameter ≤ 2.5 μm (PM_2.5_)] averaged over 1–7 days and measures of C-reactive protein (CRP), interleukin-6 (IL-6), and white blood cells (WBCs). Effect modification was investigated for diabetes, obesity, hypertension, and elevated mean inflammatory markers. We found positive associations between longer moving averages of PM_2.5_ and WBCs across all participants, with a 5.5% [95% confidence interval (CI), 0.10 to 11%] increase per interquartile increase (5.4 μg/m^3^) of PM_2.5_ averaged over the previous week. PM_2.5_ and CRP also exhibited positive associations among all individuals for averages longer than 1 day, with the largest associations for persons with diabetes, obesity, and hypertension. For example, an interquartile increase in the 5-day mean PM_2.5_ (6.1 μg/m^3^) was associated with a 14% increase in CRP (95% CI, −5.4 to 37%) for all individuals and an 81% (95% CI, 21 to 172%) increase for persons with diabetes, obesity, and hypertension. Persons with diabetes, obesity, and hypertension also exhibited positive associations between PM_2.5_ and IL-6. Individuals with elevated mean inflammatory markers exhibited enhanced associations with CRP, IL-6, and WBCs. We found modest positive associations between PM_2.5_ and indicators of systemic inflammation, with larger associations suggested for individuals with diabetes, obesity, hypertension, and elevated mean inflammatory markers.

Substantial epidemiologic evidence links particulate air pollution to adverse acute cardiovascular health effects. Associations are generally consistent across studies and robust to adjustment by smoking, weather, and seasonality (Brook et al. 2004). Although the biologic mechanisms behind these associations remain uncertain, several investigators have hypothesized that oxidative stress in the lungs from inhaled particulate matter (PM) leads to a systemic inflammatory cascade that can increase cardiovascular risk among susceptible individuals. This elevated risk can occur via increased coagulability of the blood (Seaton et al. 1995) or development and destabilization of atherosclerotic plaques (Donaldson et al. 2001).

The hypothesized role of inflammation in PM-mediated toxicity is well supported by past findings. Researchers have associated PM with influxes of inflammatory cells into the lungs (Ghio et al. 2000), enhanced production of proinflammatory cytokines by alveolar macrophages (van Eeden et al. 2001), elevated systemic blood viscosity (Pekkanen et al. 2000; Peters et al. 1997), and increased production of inflammatory cells by bone marrow (Tan et al. 2000). Acute peripheral artery narrowing (Brook et al. 2002), arterial reactivity (O’Neill et al. 2005), extent of atherosclerotic lesions (Kunzli et al. 2005; Suwa et al. 2002), and elevated risk of myocardial infarctions (Peters et al. 2001a; Zanobetti and Schwartz 2005) also have been associated with PM. In addition, relationships have been found between PM and C-reactive protein (CRP) (Peters et al. 2001b; Pope et al. 2004; Riediker et al. 2004; Seaton et al. 1999), an inflammatory marker that has been shown to be predictive of cardiovascular disease (Danesh et al. 2004; Ridker et al. 2000).

Past investigations also have demonstrated that the cardiovascular impacts of air pollution are not the same for all individuals. Enhanced susceptibility for air pollution–related cardiovascular events has been shown for older individuals and persons with conditions associated with chronic inflammation such as diabetes, coronary artery disease, and past myocardial infarctions (Bateson and Schwartz 2004; Goldberg et al. 2001; Zanobetti and Schwartz 2002). Based on these findings, it is conceivable that the short-term effects of PM on inflammation also may be enhanced among individuals with existing inflammation. Little has been published to answer this question, however. Therefore, the goals of this analysis were to evaluate short-term associations between ambient PM and markers of systemic inflammation in older adults and to explore susceptibility by conditions linked to chronic inflammation such as diabetes, obesity, and hypertension (Libby 2002).

## Materials and Methods

### Study population

Data were collected from 44 nonsmoking seniors (≥ 60 years of age) between March and June of 2002 under the supervision of the Harvard School of Public Health Human Subjects Committee. All participants were independently mobile and lived in one of four independent senior residences in suburban St. Louis, Missouri. Individuals with atrial flutter, atrial fibrillation, and/or a paced rhythm were excluded from participation because heart rate variability also was assessed during this investigation. Similarly, participants with left bundle branch blocks were selected only if their heart rate variability could be ascertained. Individuals with unstable angina and persons who were unable to provide informed written consent also were excluded from participation.

### Study design

Data for this analysis were collected as part of a more comprehensive investigation designed to examine the cardiovascular health effects of traffic-related pollution. The main goal of this investigation, which included continuous electrocardiogram measurements, was to evaluate if cumulative exposures to fresh traffic-related pollution or even moment-to-moment changes in traffic pollution would influence autonomic function in the elderly. To meet this objective, we asked a group of senior adults to participate in a series of four group trips into St. Louis for a brief activity and lunch. All trips ranged from approximately 0945 hr to 1430 hr and included two 1-hr rides aboard a diesel-powered shuttle bus. Subjects participated approximately once per month along with two to seven other individuals from their residence facility, resulting in a total of 25 trips.

A second aim of this study was to examine associations between fresh traffic-related pollution and ambient pollution on markers of inflammation. As part of this exploratory investigation, we collected venous blood samples at approximately 0900 hr on the morning after each trip. Questionnaires regarding medication and vitamin use, food consumption, and health status were also administered at that time. Blood pressure, height, and weight data were collected before participation in any trips.

### Blood analyses

Venous blood was collected per participant per trip. Each sample was analyzed promptly for white blood cells (WBCs) at the Barnes Jewish Hospital using a Coulter Ge-S system (Beckman Coulter Inc., Fullerton, CA). The remaining plasma was extracted by the Washington University Core Laboratory for Clinical Studies (St. Louis, MO), preserved at −80°C, and shipped to the Clinical and Epidemiologic Research Laboratory at Boston Children’s Hospital. Samples stored with sodium citrate were analyzed for CRP using immunoturbidimetric assays on the Hitachi 917 Chemistry Analyzer (Roche Diagnostics, Indianapolis, IN) with reagents and calibrations from Denka Seiken (Niigata, Japan). Interleukin-6 (IL-6) was analyzed using enzyme-linked immunosorbent assays from R&D Systems (Minneapolis, MN). Only samples with sufficient blood for all three assays were included in our analysis.

### Exposure measurements

Ambient PM data were obtained from the U.S. Environmental Protection Agency (EPA)–funded Supersite in East St. Louis, Illinois. Concentrations of PM with aerodynamic diameter ≤ 2.5 μm (PM_2.5_) were recorded using a continuous ambient mass monitor (Andersen Instruments; Smyrna, GA) with a Nafion diffusion dryer (Perma Pure, Toms River, NJ). Ambient black carbon (BC) was reported using a aethalometer (McGee Scientific, Berkeley, CA).

Additional measurements of group-level PM_2.5_ were collected during the 48 hr preceding each blood draw in order to better capture participants’ true exposures. These samples were collected continuously from participants’ microenvironments using a portable cart that followed subjects from a centrally located area in their living facility, onto the bus, to the activity and lunch, and finally back to their housing facility again. Installed on these carts were a DustTrak 8520 aerosol monitor (TSI Inc., Shoreview, MN) and Nafion diffusion drier. A Harvard Impactor (Air Diagnostics Environmental Inc., Harrison, ME) also was positioned on the carts as a means to calibrate the DustTrak samples because the DustTrak has been shown to overestimate concentrations despite being well correlated with several reference methods (Chang et al. 2001).

Gaseous criteria pollutant data were obtained from the Missouri Department of Natural Resources and the Illinois Environmental Protection Agency monitoring station, located immediately adjacent to the Supersite. These ambient measurements were collected using a TEI 48 analyzer (Thermo Environmental Instruments, Franklin, MA) for carbon monoxide, an API 200A analyzer (Teledyne, San Diego, CA) for nitrogen dioxide, a Dasibi 4108 analyzer (Dasibi, Glendale, CA) for sulfur dioxide, and a Dasibi 1008RS analyzer for ozone. Meteorologic parameters also were obtained from the Missouri Department of Natural Resources station and used to calculate ambient apparent temperature, a biologic weather stress index (O’Neill et al. 2003). Indoor apparent temperature was calculated using data from a HOBO data logger (Onset Computer, Bourne, MA) in the participants’ microenvironments. Daily mold and pollen data were obtained from the county health department and examined as total counts.

### Statistical analysis

We used linear mixed models (SAS, version 8.02; SAS Institute Inc., Cary, NC) to evaluate relationships between air pollution and markers of inflammation. To account for correlation among the multiple measurements collected per person, our models included random intercepts for each subject. Although autoregressive terms were evaluated, they were ultimately not used because likelihood ratio tests indicated that they were unnecessary. Random slopes were not considered because a maximum of four samples was collected from any one individual.

Exposures were evaluated in our statistical models using ambient pollutant concentrations averaged over the 1–7 days preceding each blood draw. These averaging times were calculated using hourly pollution data measured at the St. Louis Supersite and were selected based on the findings of past investigations, which found associations with indicators of inflammation on the order of days to 1 week (Peters et al. 2001b; Seaton et al. 1999). Although our main analysis focused on moving averages, we also assessed the impact of lagged ambient concentrations ranging from 1 to 7 days to confirm our findings. In addition, models were run using microenvironmental PM_2.5_ measurements averaged over the 1 and 2 days before the health measurements to examine the effects of measurement error on resulting effect estimates. (Longer averaging times for microenvironmental exposures could not be examined because they were measured only during the 2 days before the blood draws.)

Before statistical modeling, all outcome variables were transformed logarithmically because each was highly skewed. Univariate models were then run to examine the impact of various individual characteristics on the outcomes. Next, single-pollutant models for each moving average were used to evaluate ambient PM_2.5_ as a potential predictor of each inflammatory marker. Additional models containing a moving average of ambient BC, NO_2_, SO_2_, O_3_, or microenvironmental PM_2.5_ were run to confirm our ambient PM_2.5_ results. Due to limited samples and our interest in effect modification, multipollutant models were generally not employed because of power constraints. Effect estimates from our models and their 95% confidence intervals (CIs) were transformed into percent changes and reported per interquartile range (IQR) of a pollutant.

All models were controlled for sex, obesity, diabetes, and smoking history (ever/never). Time-varying parameters considered as potential confounders included apparent temperature, hour, day, trip (a proxy of activity and season), residence, mold, pollen, illness, and juice intake. Medication and vitamin consumption on the day of the blood draw was also examined, with specific focus on medications that might influence inflammation or oxidative stress (i.e., statins, inhaled steroids, aspirin, ibuprofen, and vitamins). Respiratory medications other than inhaled steroids were not examined because of insufficient daily variation. Of all the potential confounders evaluated, we selected ambient and microenvironmental apparent temperature, mold, pollen, vitamins, trip, and hour for our models based on a significant relationship with one of the outcomes at the 0.2 level among the unexposed (lower 50th percentile by exposure) (Mickey and Greenland 1989). Trip, hour, and vitamins were included in our models as categorical terms. All other variables were modeled as linear except for mold, which was treated as a linear spline with one knot for WBCs. These parameterizations were selected using loess smoothing in S-Plus 2000 (MathSoft, Cambridge, MA) as well as likelihood ratio tests and Akaike information criterion comparisons in SAS. Residual checks confirmed the parameterization of our models, and sensitivity analyses indicated that our findings were qualitatively robust to confounder selection.

Effect modification by conditions linked to chronic inflammation was investigated using interaction terms for diabetes, obesity, and hypertension. An indicator for concurrent diabetes, obesity, and hypertension was also created. For this analysis, we defined obesity by a body mass index ≥ 30 kg/m^2^ and diabetes by report of a doctor diagnosis or use of diabetes medications. Hypertension was defined as a systolic blood pressure ≥140 mm Hg, diastolic blood pressure ≥ 90 mm Hg, and/or taking hypertensive medications.

A confirmatory analysis also was conducted to compare individuals with elevated mean inflammatory markers throughout the study to the rest of the population. Because baseline measurements were unavailable, individuals were classified in the elevated marker group if their mean CRP, IL-6, or WBC levels across all samples were within the top 25th percentile of the study population. In absolute terms, these categories were defined as having a mean CRP concentration > 5.7 mg/L, a mean IL-6 concentration > 4.6 pg/mL, or a mean WBC concentration > 7.7 × 10^9^/L. Indicators were created for each inflammatory outcome individually. Effect modification by statin therapy also was examined.

## Results

### Inflammatory markers

In total, 25 group trips were conducted over the duration of the study, with most (35 of 44) subjects participating in each of their four trips. Of the 158 completed person-trips, we obtained 133 samples with sufficient volume for complete laboratory analysis. Samples were predominantly missing because of the lack of a phlebotomist or insufficient blood volume collected.

Overall summary statistics for the CRP, IL-6, and WBC levels of our samples are presented by subject characteristics in Table 1. Our 44 participants were predominantly white females with a median age of 80 years. In general, higher median levels of inflammation across our repeated samples were observed among individuals with diabetes, obesity, and/or hypertension than those without these conditions, although few of these differences were significant at the 95% confidence level. Similarly, median levels of inflammatory markers were slightly lower among those on statin therapy but not statistically different than those who were not.

### Exposure parameters

Table 2 summarizes the mean air pollution, pollen, mold, and apparent temperature levels for the day preceding each blood draw. Longer moving averages exhibited similar means but smaller ranges and standard deviations. Mean microenvironmental concentrations of PM_2.5_ were generally lower than ambient levels, although the range of values was somewhat comparable. Microenvironmental apparent temperature was more moderate and less variable than the apparent temperature outdoors. All pollutants were measured at levels well below current U.S. National Ambient Air Quality Standards (U.S. EPA 2006).

Daily concentrations of ambient PM_2.5_ and BC were positively correlated in both spring (*r* = 0.6) and summer (*r* = 0.7), whereas microenvironmental PM_2.5_ was only positively correlated with daily ambient PM_2.5_ and BC during the summer (*r* = 0.73 and *r* = 0.30, respectively). Higher summertime correlations between the ambient and microenvironmental measurements corresponded to an increased frequency of open windows during the summer. Seasonal variation also was observed for O_3_, a marker for regional pollution, which exhibited positive correlations with daily ambient PM_2.5_ during the summer (*r* = 0.2) but inverse correlations during the spring (*r* = −0.7).

### Ambient PM_2.5_ and inflammation

**Figure 1:**
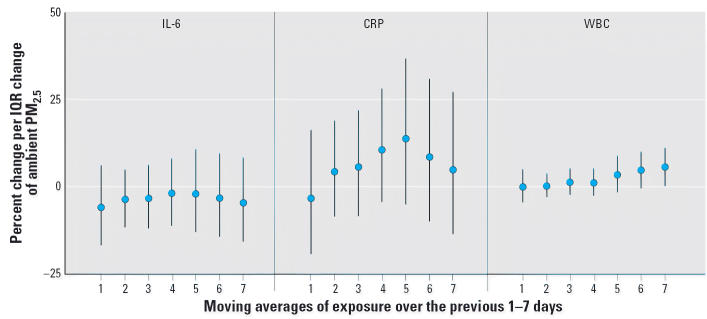
Overall associations between ambient PM_2.5_ and markers of inflammation. All models were adjusted for sex, diabetes, obesity, smoking history, ambient and microenvironmental apparent temperature, trip, pollen, mold, hour, and vitamin use. IQRs for PM_2.5_ were 10, 7.7, 7.2, 5.2, 6.1, 5.3, and 5.4 μg/m^3^ for the 1, 2-, 3-, 4-, 5-, 6-, and 7-day moving averages, respectively. Error bars indicate 95% CIs.

WBC counts were positively associated with ambient PM_2.5_ across the whole population. These associations increased with longer moving averages and reached statistical significance with the 7-day mean (Figure 1), where an IQR increase in PM_2.5_ of 5.4 μg/m^3^ was associated with a 5.5% increase in WBC counts (95% CI, 0.10–11). Associations with WBC counts remained significantly elevated through the 14-day mean (data not shown) but declined with longer moving averages. Ambient PM_2.5_ also was associated with CRP across the entire population with positive but nonsignificant relationships. These relationships were observed for all moving averages longer than 1 day and peaked with the 5-day mean. For IL-6, nonsignificant negative associations with ambient PM_2.5_ were observed among the population as a whole. Analyses of pollution lagged by 1–7 days were consistent with these findings. Similarly, associations with microenvironmental PM_2.5_ averaged over the previous 1 and 2 days demonstrated similar, although often larger, associations with each of the outcomes. (Longer averaging times for microenvironmental concentrations could not be examined, given that they were only measured during the 2 days before the blood draws.)

### Effect modification by conditions linked to inflammation

**Figure 2:**
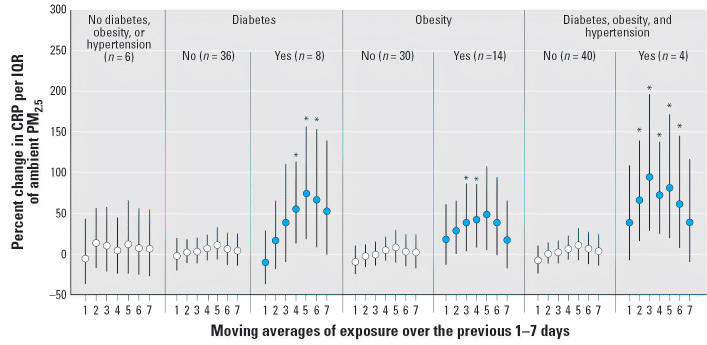
Effect modification of associations between ambient PM_2.5_ and CRP by risk factor (*n* = number of individuals). All models were adjusted for sex, diabetes, obesity, smoking history, ambient and microenvironmental apparent temperature, trip, pollen, mold, hour, and vitamin use. Error bars indicate 95% CIs. *Statistically significant interaction at the 95% confidence level.

**Figure 3:**
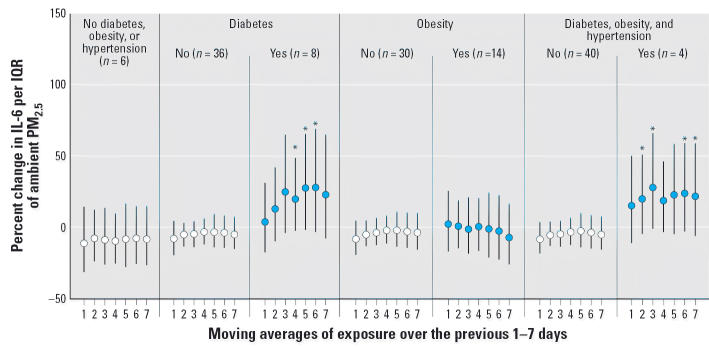
Effect modification of associations between ambient PM_2.5_ and IL-6 by risk factor (*n* = number of individuals). All models were adjusted for sex, diabetes, obesity, smoking history, ambient and microenvironmental apparent temperature, trip, pollen, mold, hour, and vitamin use. Error bars indicate 95% CIs. *Statistically significant interaction at the 95% confidence level.

**Figure 4:**
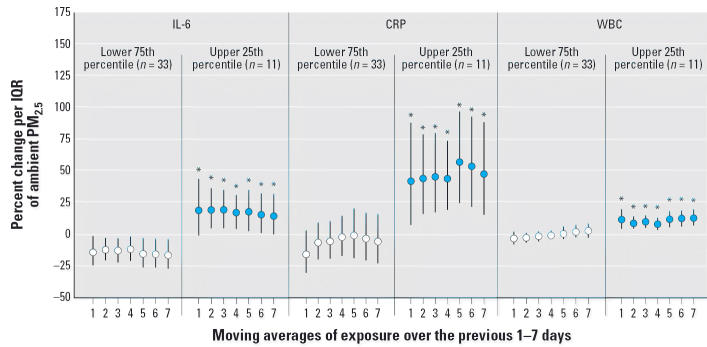
Effect modification of associations between ambient PM_2.5_ and markers of inflammation by mean inflammatory level (*n* = number of individuals). All models were adjusted for sex, diabetes, obesity, smoking history, ambient and microenvironmental apparent temperature, trip, pollen, mold, hour, and vitamin use. Error bars indicate 95% CIs. *Statistically significant interaction at the 95% confidence level.

Evidence of effect modification was most frequently observed with CRP. Associations between PM_2.5_ and CRP were consistently, and often significantly, elevated among the 8 individuals with diabetes (26 repeated samples), 14 individuals with obesity (41 repeated samples), and 4 individuals with concurrent diabetes, obesity, and hypertension (14 repeated samples) (Figure 2). For example, an IQR (6.1 μg/m^3^) increase in the 5-day mean PM_2.5_ was associated with a 48% increase (95% CI, 5.3–109) in CRP for persons with obesity, a 74% increase (95% CI, 18–158) for persons with diabetes, and an 81% increase (95% CI, 21–172) in CRP for persons with diabetes, obesity, and hypertension compared with a 12% increase (95% CI, –25 to 67) for individuals without any of these conditions. Individuals with diabetes and those with concurrent diabetes, obesity, and hypertension also demonstrated larger associations between PM_2.5_ and IL-6 (Figure 3). No evidence of effect modification was observed among the 36 individuals with hypertension, nor did any of the conditions evaluated significantly modify WBC counts (data not shown). Individuals within the top quartile of mean circulating inflammatory markers also showed larger increases in repeated measures of CRP, IL-6, and WBCs with increases in ambient PM_2.5_ (Figure 4). Identical results were produced if the median inflammatory level was used to define individuals with elevated inflammatory levels. Conversely, the 10 individuals on anti-inflammatory statin therapy demonstrated nonsignificant reductions in the association between ambient PM_2.5_ and CRP compared with those not on statin therapy.

### Other ambient pollution metrics and inflammation

Individuals with concurrent diabetes, obesity, and hypertension exhibited large positive associations between ambient BC, NO_2_, and O_3_ and CRP and IL-6, as shown for the 5-day moving average concentrations in Table 3. These associations exhibited similar patterns to those for ambient PM_2.5_ at all moving averages. WBC counts generally increased with IQR changes in ambient BC, CO, and NO_2_, whereas inverse associations were observed between WBC counts and O_3_. Inconsistent findings were observed with SO_2_.

## Discussion

In this investigation, we found evidence of positive associations between air pollution and indicators of systemic inflammation (e.g., WBCs, CRP, and IL-6) in older adults. These findings support the hypothesis that systemic inflammation is a pathway through which airborne PM leads to short-term increases in cardiac risk. We also found that the associations with CRP and IL-6 were strongest and most consistent for the 8 individuals with diabetes, 14 individuals with obesity, and 4 individuals with concurrent diabetes, obesity, and hypertension, suggesting that individuals with conditions often associated with both chronic inflammation and increased cardiac risk (Libby 2002) may be more vulnerable to the short-term proinflammatory effects of air pollution. This hypothesis is supported by the finding that individuals with the highest mean or median levels of inflammatory markers also had larger associations between air pollution and CRP, IL-6, and WBC counts.

For all three outcomes investigated, associations were strongest with PM_2.5_. Consistent associations were also generally seen for ambient BC and NO_2_, indicating that motor vehicles may be an important source for PM-mediated inflammation. Although inconsistent associations were observed between ambient CO (another marker of traffic pollution) and inflammation, this may be due to measurement error because 95% of the hourly CO concentrations were lower than the 1 ppm sensitivity of the reference method (Cogan and Lobert 1998). O_3_ and/or regional pollution may also be partly responsible for the observed relationships because positive associations were found between ambient O_3_ and CRP and IL-6. Because these relationships were generally not sensitive to stratification by season, confounding by PM_2.5_ is not likely. Associations between O_3_ and WBC counts, on the other hand, did change sign by season, corresponding to the seasonal change in the correlation between O_3_ and PM_2.5_. This suggests that there may be confounding between PM_2.5_ and O_3_ with respect to WBCs.

Because our reported associations were predominantly for ambient concentrations of air pollution, there may be some level of measurement error that is inherent to this investigation. We do not believe that this error is likely to be substantial, however, because moderately strong correlations were observed between daily concentrations of PM_2.5_ measured at ambient and microenvironmental monitors, and these correlations should increase with longer averaging periods. Strong correlations across space also have been previously reported in this region for PM_2.5_ from both sulfate and motor vehicles (Kim et al. 2005). In addition, our results are supported by the finding of similar associations with ambient concentrations and concentrations of PM_2.5_ measured in participants’ microenvironments over the time periods when both data were available.

Although our findings suggest that traffic may be an important source type for the inflammatory effects of air pollution, it is unlikely that our bus trips are responsible for the observed associations because the critical averaging periods were on the order of days rather than hours. Because bus periods lasted only 2 hr, the contribution of the bus to exposures averaged over several days was small compared with that of ambient pollution. In fact, when our analysis was limited to exposures that occurred only on the bus, the effect estimates were similar in direction to our main analysis but were smaller in magnitude, likely due to greater measurement error. Nevertheless, we cannot exclude the possibility that a specific inflammatory effect of the bus might have occurred in addition to the overall cumulative effects of ambient pollution. Such an effect might have been seen if we had measured markers of inflammation immediately before and after each trip, but we were unable to collect samples for reasons of feasibility and acceptability to our participants. We do not believe this to be a critical flaw, however, because we have less reason to suspect that pollution would have near-immediate associations with our inflammatory markers, given that other investigations of outcomes related to inflammation demonstrated associations on the order of days to a week (O’Neill et al. 2005; Peters et al. 2001b; Seaton et al. 1999; Zanobetti et al. 2004).

In addition to the timing of our associations, our results are generally consistent with other investigations with respect to directionality and magnitude of the associations for each of our inflammatory markers. For example, several past investigations have illustrated positive associations between ambient PM and CRP in the blood of older adults (Peters et al. 2001b; Pope et al. 2004; Seaton et al. 1999). Seaton et al. (1999) reported a 9.5% increase in CRP per 10 μg/m^3^ in the 3-day mean ambient PM_10_. This was comparable with our findings of an 11% increase per 10μg/m^3^ in the 5-day mean PM_2.5_ for individuals without concurrent diabetes, obesity, and hypertension. Similarly, the timing of the association between PM and CRP for our study was consistent with past work as the Monitoring of Trends and Determinants in Cardiovascular Disease (MONICA) study also reported its maximum effect with the 5-day mean (Peters et al. 2001b). Interestingly, our findings among seniors with low susceptibility also were similar to associations reported for a cohort of young policemen who exhibited a 21–32% change in CRP per 10 μg/m^3^ increase in mean PM_2.5_ over their 9-hr shift (Riediker et al. 2004).

Only one study of 30 young Singaporean national guardsmen has reported statistically significant associations between air pollution and IL-6 in blood (van Eeden et al. 2001). Other investigations have generally reported null associations between air pollution and IL-6, in agreement with our study population as a whole (Ghio et al. 2000; Nightingale et al. 2000). Despite this general lack of findings, elevated IL-6 has been found in human sputum (Nordenhall et al. 2000) and alveolar macrophages (Becker et al. 1996; van Eeden et al. 2001) after exposures to PM. In fact, PM from St. Louis was found to induce IL-6 from human alveolar macrophages with greater potency than diesel, silicon dioxide, and latex particles (Becker et al. 1996). Our findings also are supported by the fact that our associations with CRP are generally consistent with, and may lag those of, its precursor (IL-6) among individuals with diabetes, obesity, and hypertension.

Inconclusive evidence links short-term changes in WBC counts to air pollution exposures. Two investigations that found significant results for WBC counts had opposite findings (Ghio et al. 2003; Schwartz 2001), and other investigations have reported null associations (Holgate et al. 2003; Pope et al. 2004; Seaton et al. 1999). Because all of these investigations examined associations with pollution for durations < 3 days, it is possible that associations would become more consistent with longer averaging periods. Alternatively, as a composite of various cell types, WBC counts may not perform well as an indicator of the inflammatory effects of air pollution. Finally, it is possible that the response of WBCs to PM may be best measured locally in the lung rather than systemically.

Although our main results are consistent with other investigations, this study is novel in that it suggests that individuals with conditions associated with chronic inflammation may have an increased short-term inflammatory response to air pollution. One important limitation of this investigation, however, is the small number of individuals studied. In our most susceptible group, only 4 individuals with 14 measurements were classified as having concurrent diabetes, obesity, and hypertension. Despite these low numbers, our findings withstood several sensitivity checks. For example, our diabetes and obesity findings for 8 and 14 individuals, respectively, were robust to the exclusion of individuals with concurrent conditions. Similarly, no one individual dominated our results for persons with concurrent diabetes, obesity, and hypertension, and individuals without any of these three conditions demonstrated lower associations with air pollution. Our overall findings also were robust to various modeling strategies, including the use of different confounders and the use of fixed effects for each subject. In addition, confirmatory analyses supported the suggestion of susceptibility by inflammatory status, because individuals with higher levels of mean inflammatory markers demonstrated elevated associations with pollution. Similarly, we found a suggestion of reduced associations between air pollution and CRP with use of anti-inflammatory statins. Despite these assurances, future research is still needed to confirm our findings of effect modification.

To our knowledge, few investigations have attempted to investigate effect modification of short-term associations between air pollution and inflammation by chronic inflammation directly. One study, conducted in England, evaluated the influence of air pollution on the odds of myocardial infarction or stroke based on baseline levels of fibrinogen. Their findings implied that individuals with elevated inflammation were marginally more susceptible than normal individuals on high-pollution days (Prescott et al. 2000). Another study demonstrated that asthmatic children who were not on anti-inflammatory medications exhibited stronger associations between respiratory symptoms and PM_10_ than did those on anti-inflammatory medications (Delfino et al. 2002).

Although our investigation is suggestive that chronic inflammation leads to an enhanced vulnerability to the short-term inflammatory effects of ambient PM, other interconnected aspects of diabetes, obesity, and hypertension may also contribute to the susceptibility of individuals with these disease states. Possible alternative mechanisms include enhanced insulin resistance, hyperglycemia, oxidative stress, and endothelial dysfunction (Brunner et al. 2005; Libby 2002; O’Neill et al. 2005). These factors may play a role in this study given that some but not all of our diabetic, obese, and hypertensive participants had measurable manifestations of a chronic inflammatory state.

Overall, the findings of this investigation may have important implications for the biologic mechanism of air pollution and possibly its clinical relevance, because inflammation plays an important role in atherosclerosis and cardiovascular disease (Libby 2002). Chronic levels of both CRP and IL-6 have been identified as important risk factors for adverse cardiovascular outcomes (Danesh et al. 2004; Luc et al. 2003; Ridker et al. 2000). Although short-term changes in inflammation are less well understood and may differ from chronic changes, our findings still offer a potential explanation as to why individuals with diabetes, coronary artery disease, and past myocardial infarctions have elevated cardiovascular risk after acute exposures to air pollution (Bateson and Schwartz 2004; Goldberg et al. 2001; Libby 2002; O’Neill et al. 2005; Zanobetti and Schwartz 2002). It also raises the possibility that there is an interaction between the short- and long-term effects of air pollution, given that previous investigations have linked elevated exposures to PM to the development of chronic inflammatory conditions such as asthma and atherosclerosis (Delfino 2002; Suwa et al. 2002).

In summary, our data suggest that increases in air pollution may be associated with increases in systemic inflammation in older adults. Associations between pollution and short-term increases in inflammatory markers were the strongest for individuals with diabetes; obesity; and concurrent diabetes, obesity, and hypertension. Elevated associations were also found among persons with higher mean levels of inflammatory markers. Such findings support the hypothesis that the cardiovascular effects of air pollution are partially mediated via inflammation and suggest that individuals with existing inflammation and existing cardiac risk factors may be especially susceptible to the inflammatory effects of air pollution.

## Correction

In Table 1 of the manuscript published online, the ranges of CRP for both “Yes” and “No” under “Statin therapy” were incorrect. These values have been corrected here.

## Figures and Tables

**Table 1:** Levels of inflammatory markers by participant characteristics.

			Median (range)		
	Subjects [*n* (%)]	Samples (*n*)	CRP (mg/L)	IL-6 (pg/mL)	WBCs (× 10^9^/L)
All participants	44 (100)	133	2.2 (0.25–41)	2.9 (0.98–18)	6.4 (3.4–11)
Sex					
Female	36 (82)	107	2.8 (0.25–41)	2.9 (0.98–18)	6.7 (3.4–11)
Male	8 (18)	26	1.1 (0.27–15)	3.0 (1.0–14)	5.6 (3.9–8.4)
Race					
White	41 (93)	125	2.5 (0.25–38)	2.9 (1.0–18)	6.4 (3.4–11)
African American	3 (7)	8	1.2 (0.69–41)	2.7 (0.98–5.1)	4.8 (4.3–6.8)
Age (years)					
60–79	19 (43)	56	3.0 (0.26–41)	2.8 (0.98–18)	6.4 (4.5–9.0)
80–95	25 (57)	77	1.7 (0.25–38)	2.9 (1.0–14)	6.3 (3.4–11)
Cigarette smoking					
Former	21 (48)	63	2.7 (0.25–41)	3.4 (0.98–18)*	6.7 (3.9–10)
Never	23 (52)	70	2.2 (0.26–19)	2.7 (1.0–14)*	6.1 (3.4–11)
Diabetes					
Yes	8 (18)	26	3.3 (0.27–19)	2.7 (1.0–7.2)	7.2 (4.3–11)
No	36 (82)	107	2.1 (0.25–41)	2.9 (0.98–18)	6.3 (3.4–11)
Obesity					
Yes	14 (32)	41	4.8 (0.66–41)	3.0 (0.98–7.2)	7.2 (4.6–11)*
No	30 (68)	92	1.7 (0.25–38)	2.7 (1.0–18)	6.0 (3.4–9.4)*
Hypertension					
Yes	36 (82)	108	2.6 (0.25–41)	3.0 (0.98–18)	6.6 (3.9–11)*
No	8 (18)	25	1.8 (0.26–6.8)	1.9 (1.1–6.1)	5.4 (3.4–8.1)*
Diabetes with obesity and hypertension					
Yes	4 (9)	14	5.4 (0.66–19)	3.8 (2.1–7.2)	8.2 (4.6–11)
No	40 (91)	119	1.9 (0.25–41)	2.7 (0.98–18)	6.3 (3.4–11)
Statin therapy					
Yes	10 (23)	33	2.2 (0.025–6.8)	2.7 (1.2–14)	6.3 (4.7–8.5)
No	34 (77)	100	2.4 (0.026–41)	2.9 (0.98–18)	6.5 (3.4–11)

* Groups were significantly different at the 0.05 level in a univariate model with random subject effects.

**Table 2:** Descriptive statistics for pollution, biologic, and meteorologic variables (1-day averages).

			Quartile			
	No. samples	Mean ± SD	0th	25th	75th	100th
Ambient						
PM_2.5_ (μg/m^3^)	24	16 ± 6.0	6.5	12	22	28
BC (ng/m^3^)	24	900 ± 280	290	730	1,100	1,400
CO (ppm)	22	0.43 ± 0.13	0.096	0.38	0.49	0.66
NO_2_ (ppb)	21	17 ± 3.3	11	15	19	22
SO_2_ (ppb)	23	6.7 ± 8.2	1.2	2.1	7.4	27
O_3_ (ppb)	22	24 ± 9.4	1.2	17	29	44
Mold counts (pt/m^3^)	24	18,000 ± 20,000	1,000	3,500	27,000	68,000
Pollen counts (pt/m^3^)	24	480 ± 1,000	6.00	44	390	4,600
Apparent temperature (°C)^a^	24	17 ± 9.7	−2.5	9.2	24	31
Microenvironmental						
PM_2.5_ (μg/m^3^)	23	9.8 ± 4.5	3.5	7.1	11	22
Apparent temperature (°C)^a^	24	23 ± 2.1	19	21	23	27

A maximum of 24 trips was included in this analysis because data from one trip was lost due to last-minute cancellation by our phlebotomist.

a Apparent temperature was calculated by −2.653 + (0.994 × temperature in °C) + [0.0153 × (dew point temperature in °C)^2^] (O’Neill et al. 2003).

**Table 3:** Associations (95% CIs) between 5-day mean ambient pollutant concentrations and markers of inflammation by conditions linked to inflammation.

	CRP	IL-6	WBC (× 10^9^/L)
PM_2.5_			
All	14 (−5.4 to 37)	−2.1 (−13 to 11)	3.4 (−1.8 to 8.9)
3 Conditions present	81 (21 to 172)*^,^**	23 (−5.3 to 59)	0.4 (−8.8 to 11)
≤2 Conditions present	11 (−7.3 to 33)**	−3.1 (−14 to 9.7)	3.6 (−1.7 to 9.1)
BC			
All	13 (−0.34 to 28)	−0.8 (−8.9 to 8.0)	1.3 (−2.1 to 4.8)
3 Conditions present	49 (16 to 90)*^,^**	15 (−2.2 to 35)**	0.05 (−5.9 to 6.3)
≤2 Conditions present	9.0 (−3.8 to 24)**	−2.7 (−11 to 6.2)**	1.5 (−2.0 to 5.1)
CO			
All	0.22 (−11 to 13)	−5.2 (−12 to 2.7)	2.9 (−0.22 to 6.2)
3 Conditions present	−16 (−32 to 4.3)	4.1 (−9.6 to 20)	4.2 (−1.2 to 10)
≤2 Conditions present	4.3 (−8.1 to 18)	−7.2 (−14 to 1.0)	2.6 (−0.7 to 6.0)
NO_2_			
All	15 (−11 to 49)	−2.7 (−18 to 16)	0.3 (−6.3 to 7.4)
3 Conditions present	36 (−5.2 to 94)	8.2 (−12 to 33)**	0.8 (−7.3 to 9.6)
≤2 Conditions present	12 (−14 to 45)	−6.0 (−21 to 12)**	0.3 (−6.4 to 7.4)
SO_2_			
All	−8.3 (−15 to −0.65)*	−3.8 (−8.9 to 1.5)	2.3 (0.1 to 4.5)*
3 Conditions present	−20 (−50 to 26)	12 (−17 to 50)	−0.7 (−11 to 11)
≤2 Conditions present	−8.0 (−15 to −0.24)*	−4.1 (−9.3 to 1.3)	2.3 (0.1 to 4.6)*
O_3_			
All	−3.9 (−17 to 11)	8.0 (−1.6 to 19)	−2.1 (−5.8 to 1.7)
3 Conditions present	41 (7.6 to 84)*^,^**	22 (1.9 to 45)*	−4.2 (−10 to 2.5)
≤2 Conditions present	−5.7 (−18 to 8.1)**	7.3 (−2.4 to 18)	−2.0 (−5.7 to 1.8)

All models were adjusted for sex, obesity, diabetes, smoking history, ambient and microenvironmental apparent temperature, mold, pollen, trip, hour, and vitamins. To maximize comparability, only records with complete PM_2.5_, BC, CO, SO_2_, and O_3_ data were evaluated. The NO_2_ data were too sparse to fairly limit the data set. IQRs were 6.1 μg/m^3^, 230 ng/m^3^, 0.13 ppm, 3.6 ppb, 2.3 ppb, and 7.2 ppb for PM_2.5_, BC, CO, NO_2_, SO_2_, and O_3_, respectively.

* The association met statistical significance at the 95% confidence level.

** The interaction met statistical significance at the 95% confidence level.
